# The chromosome copy number of the hyperthermophilic archaeon *Thermococcus kodakarensis* KOD1

**DOI:** 10.1007/s00792-015-0750-5

**Published:** 2015-05-08

**Authors:** Sebastiaan K. Spaans, John van der Oost, Servé W. M. Kengen

**Affiliations:** Laboratory of Microbiology, Wageningen University, PO Box 8033, 6700 EJ Wageningen, The Netherlands

**Keywords:** *Thermococcus kodakarensis*, Chromosome copy number, Genome copy number, Archaea, Polyploidy, Euryarcheaota

## Abstract

The euryarchaeon *Thermococcus kodakarensis* is a well-characterized anaerobic hyperthermophilic heterotroph and due to the availability of genetic engineering systems it has become one of the model organisms for studying Archaea. Despite this prominent role among the Euryarchaeota, no data about the ploidy level of this species is available. While polyploidy has been shown to exist in various Euryarchaeota, especially *Halobacteria*, the chromosome copy number of species belonging to one of the major orders within that phylum, i.e., the Thermococcales (including *Thermococcus* spp. and *Pyrococcus* spp.), has never been determined. This prompted us to investigate the chromosome copy number of *T. kodakarensis*. In this study, we demonstrate that *T. kodakarensis* is polyploid with a chromosome copy number that varies between 7 and 19 copies, depending on the growth phase. An apparent correlation between the presence of histones and polyploidy in Archaea is observed.

**Electronic supplementary material:**

The online version of this article (doi:10.1007/s00792-015-0750-5) contains supplementary material, which is available to authorized users.

## Introduction

*Thermococcus kodakarensis* is a marine hyperthermophilic, heterotrophic, obligate anaerobic euryarchaeon that can grow at temperatures ranging from 60 to 100 °C, with an optimum at 85 °C. *T. kodakarensis* can grow on a variety of organic substrates in the presence of elemental sulfur, producing hydrogen sulfide, or when pyruvate or starch are present, in the absence of elemental sulfur producing hydrogen. Under optimal conditions, *T. kodakarensis* grows rapidly to high cell densities, having an estimated doubling time of ~40 min. Moreover, it can be grown on solid media, forming defined but small colonies (Atomi et al. 2004; Morikawa et al. 1994). With its genome being fully sequenced (Fukui et al. 2005), its natural competence and the establishment of various genetic engineering techniques (Hileman and Santangelo 2012; Santangelo and Reeve 2011; Sato et al. 2003, 2005), *T. kodakarensis* rapidly became one of the model organisms for studying anaerobic Archaea.

While genome modifications are generally relatively easily obtained in this organism, and thus routinely applied [e.g., (Fukuda et al. 2008; Imanaka et al. 2006; Kanai et al. 2011; Santangelo et al. 2011; Sato et al. 2003, 2004; Takemasa et al. 2011; Yokooji et al. 2013)], occasionally, we experienced difficulties in obtaining transformants with two homologous recombinations. Without going into too much detail, those data suggested that the desired double crossover modification co-existed with single crossover insertions, as well as with the wild-type chromosome. The difficulty in obtaining homozygous recombinant cultures by repetitive cycles of re-streaking and screening, as well as the repeated observation of this phenomenon among different transformation attempts, suggested that *T. kodakarensis* may possess multiple copies of its chromosome.

In contrast to the general idea of prokaryotes being monoploid, polyploidy has in recent years been demonstrated for various species including many Euryarchaeota (Table 1), the phylum that *T. kodakarensis* belongs to. Although monoploid and polyploid species can co-exist within one phylum (Pecoraro et al. 2011), none of the Euryarchaeota investigated so far was monoploid. It has therefore been suggested that polyploidy might be a common feature in Euryarchaeota (Hildenbrand et al. 2011). In this study, we determined the chromosome copy number of the euryarchaeon *T. kodakarensis*. The copy number was determined for three different growth phases; the early exponential phase, the late exponential/linear phase and the early stationary phase. Our results confirm that *T. kodakarensis* is truly polyploid and that the exact ploidy level is dependent on the growth phase. In addition, a potential correlation between the presence of histones and polyploidy in Archaea is suggested.

**Table 1:** Chromosome copy number of archaeal species and the distribution of archaeal histones

Phylum Order *Species*	Doubling time (h)	Genome copy number		Ploidy	Histones	Reference
		Exp. phase	Stat. phase			
Euryarchaeota						
Halobacteriales						
*Halobacterium cutirubrum*	6.7	10.6		Polyploid	Yes^a^	Chant et al. (1986)
*Halobacterium cutirubrum*	13.3	6.3		Polyploid	Yes^a^	Chant et al. (1986)
*Halobacterium salinarum*	4	25	1	Polyploid	Yes	Breuert et al. (2006)
*Halobacterium salinarum*	8	25	15	Polyploid	Yes	Breuert et al. (2006)
*Haloferax volcanii*	4	17	10	Polyploid	Yes	Breuert et al. (2006)
*Haloferax mediterranei*		30–55	20–30	Polyploid	Yes	Liu et al. (2013)
Methanosarcinales						
*Methanosarcina acetivorans*	6	18	16	Polyploid	Yes	Hildenbrand et al. (2011)
*Methanosarcina acetivorans*	49	3	5	Polyploid	Yes	Hildenbrand et al. (2011)
Methanobacteriales						
*Methanothermobacter thermautotrophicus*		2	1–2	Diploid^b^	Yes	Majerník et al. (2005)
Methanomicrobiales						
*Methanoculleus marisnigri*					Yes	
Thermococcales						
*Thermococcus kodakarensis*	1.25	19.4	7.5	Polyploid	Yes	This study
*Pyrococcus furiosus*		Multiple			Yes	Malandrin et al. (1999)
*Pyrococcus abyssi*		Multiple			Yes	Marie et al. (1996)
Methanococcales						
*Methanocaldococcus jannaschii*	0.5	10–15	1–5	Polyploid	Yes	Malandrin et al. (1999)
*Methanococcus maripaludis*	2	55	30	Polyploid	Yes	Hildenbrand et al. (2011)
Thermoplasmatales						
*Thermoplasma acidophilum*					No^c^	
*Thermoplasma volcanium*					No^c^	
*Ferroplasma acidarmanus*					No^c^	
Archaeoglobales						
*Archaeoglobus fulgidus*		Multiple			Yes	Malandrin et al. (1999)
Methanopyrales						
*Methanopyrus kandleri*					Yes	
Methanocellales						
*Methanocella paludicola*					Yes	
Crenarchaeota						
Acidilobales						
*Acidianus hospitalis*		1–2	2	Monoploid	No	Lundgren et al. (2008)
Desulfurococcales						
*Aeropyrum pernix*	3.3	1–2	2	Monoploid	No	Lundgren et al. (2008)
Fervidicoccales						
*Fervidicoccus fontis*					No	
Sulfolobales						
*Sulfolobus acidocaldarius*	3.5	1–2	2	Monoploid	No	Bernander and Poplawski (1997)
*Sulfolobus tokodaii*	8	1–2	2	Monoploid	No	Lundgren et al. (2008)
*Sulfolobus solfataricus*	7	1–2	2	Monoploid	No	Bernander and Poplawski (1997)
Thermoproteales						
*Pyrobaculum aerophilum*		1–2	2	Monoploid	No	Lundgren et al. (2008)
*Pyrobaculum calidifontis*	3.4	1–2	2	Monoploid	No	Lundgren et al. (2008)
*Thermofilum pendens*					Yes	
*Caldivirga maquilingensis*					Yes	
*Vulcanisaeta distributa*					Yes	
Thaumarchaeota						
Cenarchaeales						
*Cenarchaeum symbiosum*					Yes	
Nitrosopumilales						
*Nitrosopumilus maritimus*					Yes	
Nitrososphaerales						
*Nitrososphaera viennensis*					Yes	
Korarchaeota						
*Candidatus* *Korarchaeum cryptofilum*					Yes	
Nanoarchaeota						
*Nanoarchaeum equitans*					Yes	

The table gives an overview of all studies in which chromosome copy numbers of archaeal species have been determined. The table was adapted from a table presented by Hillenbrand et al. (2011), by including chromosome copy numbers of several additional species, grouping the results per order and including an extra column showing the presence or absence of archaeal histone homologs. The organisms that were not present in the histone database were additionally checked by BLAST for histone homologs. For the orders for which no ploidy level data is available, representative species, whose genome is available and could be screened for the presence of archaeal histone homologs, were randomly picked

^a^The species *Halobacterium salinarum*, *Halobacterium halobium*, and *Halobacterium cutirubrum* are so similar that they should be regarded as strains of one species named *Halobacterium salinarum*. *H. cutirubrum* is therefore, as such, not present in the histone database (Ventosa and Oren 1996)

^b^The cells grow in filaments; the numbers given are genome copies per cell, not per filament

^c^The thermoplasmatales do not encode archaeal histones, however, they do encode homologs of HU-proteins (bacterial histone-like proteins)

## Materials and methods

### Culture conditions and growth analysis

**Fig. 1:**
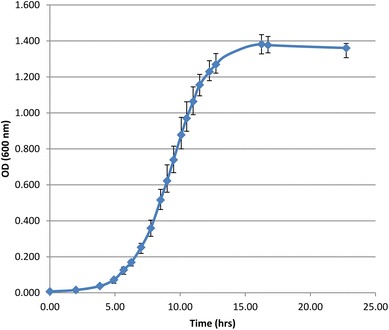
Growth curve increase in optical density of *Thermococcus kodakarensis* KOD1 (WT) in time on ASW-YT-Pyr medium. The *graph* represents the average optical density at 600 nm (OD_600_) of five independent cultures

The hyperthermophilic archaeon *Thermococcus kodakarensis* KOD1 wild-type strain was grown anaerobically at 85 °C in ASW-YT-Pyr medium. The ASW-YT medium was composed of 0.8× artificial seawater (0.8× ASW), 5.0 g/L yeast extract, 5.0 g/L tryptone and 0.8 mg/L resazurine. Before inoculation, 5.0 g/L sodium pyruvate (ASW-YT-Pyr medium) was added to the medium, as well as Na_2_S.9H_2_O, until it became colorless. For all cultivations, 120-mL serum bottles containing 40 mL culture with N_2_ as headspace were used. Cultures were continuously shaken (120 rpm) during growth. Growth was monitored by measuring the optical density at 600 nm (OD_600_) using a U-1500 spectrophotometer (Hitachi). Five independent cultures were grown, and their average OD_600_ value and their variances were calculated (Fig. 1).

### Sample extraction, cell lysis and determination of cell number

Cultures for chromosome copy number determination were inoculated from fresh pre-cultures in the late exponential growth phase (1 % inoculum) and grown to the desired optical density. For each growth phase, samples were taken from three independent cultures. When the cultures reached the desired optical density, 1.0 and 1.5 mL samples were taken by syringe and cells were harvested by centrifugation (5000*g*, 30 min, RT), for the “agarose gel method” and “real-time PCR method”, respectively. The supernatant was checked microscopically to verify that it was free of cells. Cell pellets were stored at −20 °C until further processing. The cell number of the cultures at the moment of sampling was determined by directly counting the cells using a Neubauer counting chamber. The pellets were resuspended in 100 µL TE buffer (10 mM Tris; 0.1 mM EDTA, pH 8.5). Cell lysis was performed by adding SDS to a final concentration of 0.3 %. Complete cell lysis was verified microscopically and the integrity of genomic DNA was verified by agarose gel electrophoresis.

### Preliminary quantification of chromosome copy number by “agarose gel method”

To determine if *T. kodakarensis* could indeed possess multiple chromosomes per cell, and hence if it would be worth pursuing quantification by a more accurate method, a preliminary quantification experiment was performed first. This was done by preparing cell lysates of three independent cultures having an OD_600_ of ~0.76 (see above). To minimize the disturbing effect of SDS during agarose gel electrophoresis, the cell lysate was diluted tenfold by adding TE buffer to a final volume of 1 mL. Aliquots of 5 µL of each sample were loaded, in duplicate, on a 1 % agarose gel containing the SYBR Safe DNA gel stain (Life technologies), flanked by a known amount of a 1 kb GeneRuler DNA ladder (Thermo scientific). Since the SYBR Safe DNA gel stain moves away from the migrating DNA, the stain was also added to the running buffer to ensure an equal concentration of the stain in the entire gel. After electrophoresis, the DNA concentration of each sample was quantified by measuring the fluorescence intensity of the corresponding bands using a G:box imager and GeneSys analysis software (Syngene) and comparing it to the ladder with known concentrations of DNA. The molecular weight (in Daltons) of one chromosome copy was computed with the “DNA molecular weight calculator” (http://www.currentprotocols.com/WileyCDA), which was then converted to its mass (in grams) using a “Dalton to grams conversion calculator” (http://www.unitconversion.org). Next, the chromosome copy number in the cell lysate was calculated by dividing the DNA concentration of the lysate by the mass of one chromosome. Finally, the number of chromosome copies per cell was calculated by combining this with the original cell density of the sample (supplementary file).

### Quantification of chromosome copy number by “real-time PCR method”

To determine chromosome copy numbers, a previously developed real-time PCR approach was used (Breuert et al. 2006). A schematic overview of the method can be found in Hildenbrand et al. (2011). First, to make a standard curve, a 1-kb fragment was amplified using conventional PCR with genomic DNA as template. The genomic DNA was isolated using a genomic DNA purification kit (Thermo scientific, protocol for Gram positive bacteria). The 1 kb fragment generated in this study was internal to the chitinase gene (TK1765) as the amplification of this fragment had previously been shown to be successful, resulting in a clear, single product-band on gel. The primers used are shown in Table S1. The fragment was purified using preparative agarose gel electrophoresis (0.8 %) and a GeneJET Gel extraction kit (Thermo Scientific), and concentrated by an DNA Clean and Concentrator kit (ZYMO RESEARCH). The DNA concentration was determined with a Nanodrop ND-1000, and the number of DNA molecules was calculated using the molecular weight computed with “oligo calc” (http://www.basic.northwestern.edu/biotools/oligocalc.html). A serial tenfold dilution containing defined numbers of standard molecules was generated in triplicate, and used for real-time PCR analysis in parallel with a dilution series of cell lysate samples.

To minimize the inhibiting effect of SDS on real-time PCR, the cell lysate (see above) was first diluted to a final SDS concentration of 0.03 % by adding TE buffer to a final volume of 1 mL. This diluted cell lysate sample was used for making the dilution series that was used for real-time PCR. A 5-µL aliquot of the dilution series was used as template in the real-time PCR analyses for quantification of chromosome copy numbers (see below). To ensure that the PCR efficiency of the cell lysate dilution series and the standard dilutions series was identical, the standard dilution series was also added to 4 selected dilutions of the cell lysate sample as an internal control, yielding 6 different dilution series in total. The fragment targeted in the real-time PCR analysis was 293 bp and was internal to the standard fragment.

Real-time PCR was performed in 25-µL reactions containing 5 µL of template (cell lysate, standard, or cell lysate with added standard), 100 mM of primer BG5331 and BG5332 each and 2× qPCR Master Mix (iQ SYBR Green Supermix, BIO RAD). The master mix contained antibody-mediated hot start iTaq DNA polymerase, dNTPs, SYBR GREEN I, MgCl_2_, enhancers, stabilizers and fluorescein (concentrations not released by BIO RAD). The PCR reaction conditions were 10 min at 95 °C, 40 cycles of amplification (30 s at 95 °C, 30 s at 59 °C, 30 s at 72 °C), and an final incubation of 5 min at 72 °C. The real-time PCRs were performed in a CFX96 thermal cycler (BioRad). At the end of the PCR, a melting curve was generated using the following settings: 65–95 °C with increments of 0.5 °C/5 s. For each sample the numbers of cycles was determined until its fluorescence intensity reached the threshold (*C*q value). By comparison of the threshold cycle (*C*q) differences of the different dilutions it was verified that the PCR was exponential at least up to the threshold DNA concentration used for the analysis (i.e., a tenfold dilution corresponds to a *C*q difference of 3.6 > *C*q > 3.1, having an efficiency of 90–110 %) The *C*q values of the standards were used to construct a standard curve (Fig. 3) that was used to determine the chromosome copy number in the cell lysates and to check the PCR efficiency in the cell lysates including internal standards. In combination with the cell density, the number of chromosome copies per cell was calculated.

### Method optimization

The protocol as outlined above was established by optimizing different steps individually. First, various genomic DNA isolation methods, such as sonication, French press, osmotic shock, CTAB/NaCl, lysozyme, SDS or repetitive freeze–thaw cycles were tested for their effectiveness (extent of cell lysis and gDNA recovery) and ability to leave the genomic DNA intact. The only method meeting both standards, and yielding reproducible results, was the addition of SDS to the cell suspensions. Since SDS is known to have disturbing effects on both PCR and agarose gel electrophoresis, a series of SDS concentrations was tested to determine the minimal concentration needed for complete lysis and yielding reproducible results. This was found to be 0.3 %. Chromosome copy number quantification by the “agarose gel method” was improved by addition of the SYBR Safe DNA gel stain to the running buffer to ensure an equal concentration of the stain in the entire gel. Real-time PCR was optimized by testing three different primer sets for the amplification of a small internal fragment (all ~300 bp). For each primer set, the optimal annealing temperature was determined by gradient PCR. The primer set BG5331-BG5332 (see Table S1), resulting in the amplification of a 293-bp fragment, turned out to be the most reproducible, without any artificial by-products. The latter was confirmed by visualizing the amplified fragments on a 1 % agarose gel as well as by including a melting curve in the real-time PCR protocol.

## Results

### Growth analysis and preliminary quantification by “agarose gel method”

**Fig. 2:**
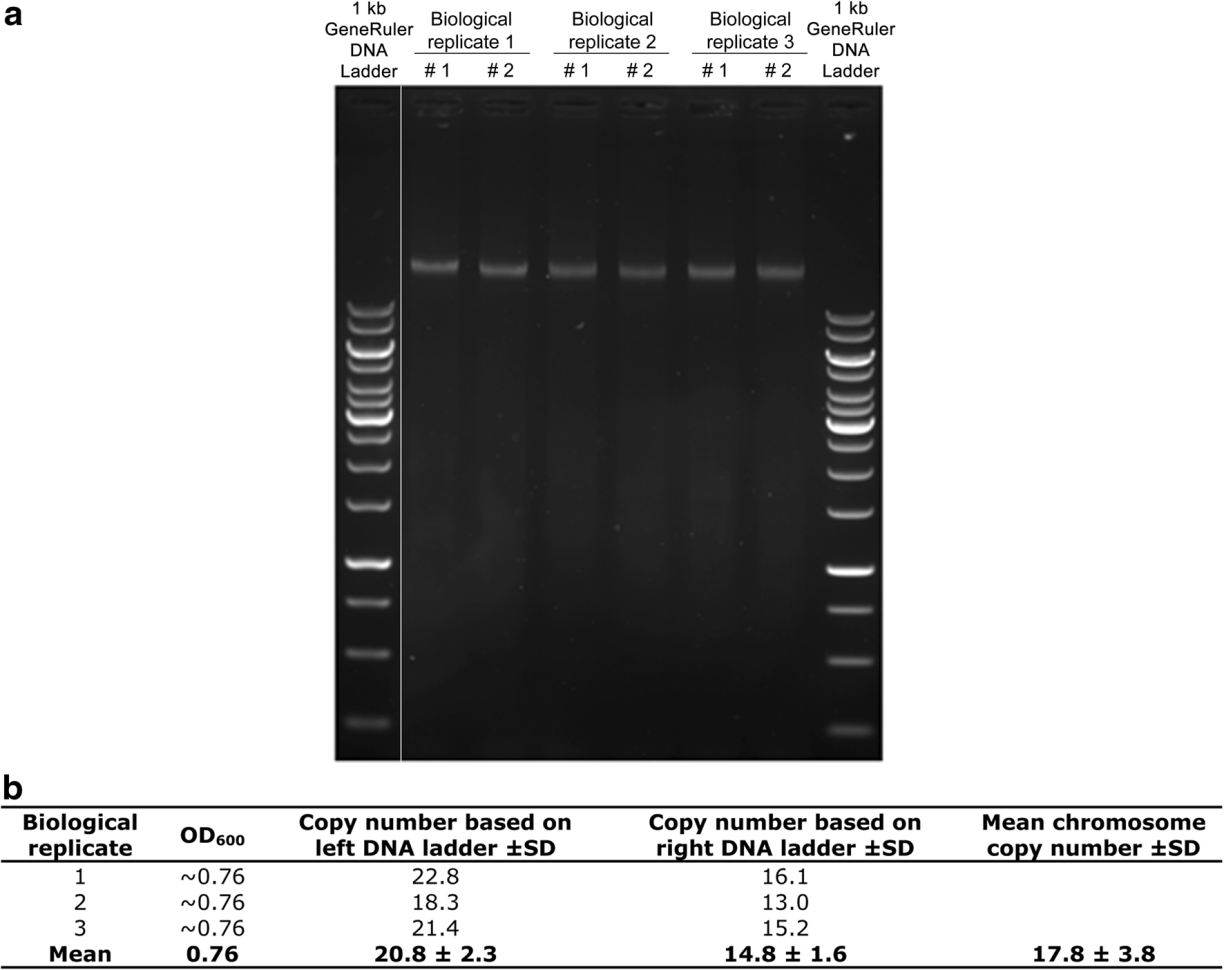
Chromosome copy number quantification by “agarose gel method”. **a** Agarose gel containing cell lysate samples of three independent cultures, in duplicate, flanked by a 1-kb GeneRuler DNA ladder. The *white line* indicates non-adjacent lanes of the same gel. The DNA concentration and chromosome copy number of each sample was quantified by comparing the fluorescence intensity of the gel bands to that of the left and right ladder. **b** The number of chromosome copies per cell. This was calculated by dividing the chromosome copy number of the lysate by the original cell density of each replicate

To make growth curves, five independent cultures of *T. kodakarensis* were grown on pyruvate, and their average OD_600_ values and their variances were used to construct Fig. 1. The results show that growth is highly reproducible, which is a prerequisite for reliable chromosome copy number quantification methods. Before an exact chromosome copy number quantification was performed, however, a preliminary in-gel quantification was applied first to determine the probability of polyploidy in *T. kodakarensis*, and hence if a more exact quantification method was worth applying. This was done by preparing cell lysate samples, in duplicate, of three independent cultures having an OD_600_ of ~0.76. Corresponding cell densities were determined and aliquots of the 6 samples obtained were loaded on an agarose gel flanked by a known amount of a 1-kb GeneRuler DNA ladder (Thermo Scientific) as shown in Fig. 2a. The DNA concentration and chromosome copy number of each sample was quantified by measuring the fluorescence intensity of the corresponding gel bands and comparing it to the ladder containing known concentrations of DNA. Together with the original cell density of each sample, the number of chromosome copies per cell was calculated (supplementary file). The results are shown in Fig. 2b. These results clearly indicate polyploidy in *T. kodakarensis*, suggesting an average chromosome copy number of 17.8 ± 3.8. It was therefore decided to determine the chromosome copy number by a more exact quantification method as well.

### Quantification by “real-time PCR method”

To certify polyploidy in *T. kodakarensis* and to quantify chromosome copy numbers more accurately, a previously developed real-time PCR method for quantification of chromosome copy numbers was optimized for *T. kodakarensis.* This chromosome quantification method has been established for haloarchaea in 2006 (Breuert et al. 2006), and has since then been applied to various other prokaryotes. The method has been validated against several independent techniques and has shown to be very reliable (Griese et al. 2011; Hildenbrand et al. 2011; Pecoraro et al. 2011). A schematic overview of the method can be found in the previous report (Hildenbrand et al. 2011).

To give a short overview, a fragment of about 1 kb is amplified first, using standard PCR with genomic DNA as template. A dilution series of this fragment is made and used to generate a standard curve in a real-time PCR analysis. This is done by amplifying a fragment of about 300 bp, internal to the standard fragment, and by plotting the threshold value (*C*q) against the corresponding template concentration (in copies/µL). To quantify the chromosome copy number of the species of interest, cells are lysed and a dilution series of the resulting cell lysates is analyzed by real-time PCR in parallel to the standards. By comparing the *C*q values of the cell lysate samples to the standard curve, the number of chromosome copies per sample can be determined, which in combination with the cell density of the corresponding culture, allows for calculating the ploidy level.

**Fig. 3:**
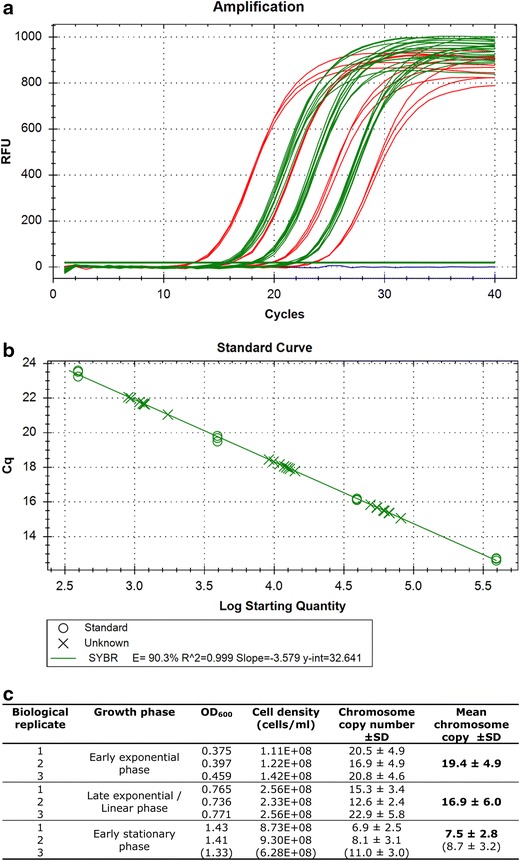
Chromosome copy number quantification by real-time PCR method. **a** Selected real-time PCR results. The fluorescent intensity curves from four standard dilutions (*in red*), three sample dilutions (*in green*), and a no-template control (NTC, *in blue*) are shown. The reactions containing the standard dilutions were performed in triplicate and the ones containing the samples in duplicate (these technical duplicates were in addition to the biological triplicates). The curves shown are the result of serial tenfold dilutions of the templates. In addition to the selected reactions shown here, each experiment included more standard and more sample dilutions. **b** Standard curve. The standard curve was created by plotting the threshold values (*C*_q_) of the individual reactions against the corresponding template concentration. **c** Average chromosome copy number values and their standard variations of *T. kodakarensis* in three different growth phases. The third sample of the early stationary phase (*in parentheses*) could be argued not to be in the early stationary phase. Therefore, the average chromosome copy number and its standard deviation in this phase was calculated by both including (*in parentheses*) and excluding the third biological replicate

Cultures for chromosome copy number determination were grown to the optical densities as shown in Fig. 3, representing the early exponential phase, late exponential/linear phase and early stationary phase. For each growth phase, samples were taken from three independent cultures. When the cultures reached the desired optical density, 1.5-mL samples were taken and cells were harvested by centrifugation and the supernatant was checked microscopically to verify that it was free of cells; which was the case. The cell numbers of the cultures at the moment of sampling were determined (see Fig. 3c) and cells were lysed by adding SDS, which was found to be the most effective (see “Materials and methods”).

A tenfold dilution series of the cell lysates was prepared and used for real-time PCR analysis in parallel with the standard fragment. To verify that the added SDS or other components present in the cell lysate did not negatively influence the reaction itself, the standard dilution series was also added to 4 selected dilutions of the cell lysate sample as an internal control. No inhibiting effects were observed for any of the selected samples. The final real-time PCR was performed by including technical duplicates, in addition to the biological triplicates, and had an efficiency of 90.3 %, an *R*^2^ of 0.999 and a slope of −3.5. The results of the real-time PCR are shown in Fig. 3. It was found that cells in the early exponential phase contain about 19.4 ± 4.9 copies of their chromosome, and that this value drops to 7.5 ± 2.8 copies in early stationary phase. The two independent quantification methods thus both confirm polyploidy in *T. kodakarensis*.

## Discussion

The results obtained in this study show that the euryarchaeon *T. kodakarensis* contains multiple chromosomes, with numbers varying from ~7 to 19 copies per cell. Although polyploidy has been demonstrated for several Euryarchaeota, the chromosome copy number of species belonging to one of the major orders within that phylum, i.e., the Thermococcales (including *Thermococcus* spp. and *Pyrococcus* spp.), has never been determined. The existence of polyploidy in *T. kodakarensis* supports the suggestion of Hildenbrand et al. (2011) that polyploidy might be a common trait of all Euryarchaeota (Hildenbrand et al. 2011). The only exception known so far is *Methanothermobacter thermautotrophicus*, which was reported to be diploid instead (Majerník et al. 2005). *T. kodakarensis* was found to have a growth-phase-dependent ploidy level having as many as 19.4 ± 4.9 chromosome copies at the early exponential phase, which is strongly decreasing towards the stationary phase. The observation of a growth-phase-dependent ploidy level has been reported for many other prokaryotes as well, including the majority of the investigated euryarchaeal species (Breuert et al. 2006; Griese et al. 2011; Hildenbrand et al. 2011; Pecoraro et al. 2011). Unfortunately, no common pattern has emerged from the characterized prokaryotic species (Breuert et al. 2006). Where some species have a higher chromosomal copy number in the exponential phase [e.g., *T. kodakarensis* or some halophilic Archaea (Breuert et al. 2006)], others have a higher chromosomal copy number in the stationary phase [e.g., *Azotobacter vinelandii* (Maldonado et al. 1994)].

Although a chromosome copy number of around 19 might seem to be rather high, this result is comparable to copy numbers (2–55) found in many other Euryarchaeota (see Table 1). The euryarchaea *Methanococcus maripaludis* and *Haloferax mediterranei* were even found to have up to 55 chromosome copies per cell, which is the highest copy number for Archaea described so far. While this is more than double the number found for *T. kodakarensis*, it is still low compared to the copy numbers reported for the cyanobacterium *Synechocystis* sp. PCC 6803 or the firmicute *Epulopiscium* spp., having up to 218 or even tens of thousands of copies, respectively (Griese et al. 2011; Mendell et al. 2008). The bacterium *Epulopiscium* spp., however, is quite extraordinary in that it is one of the largest known bacteria (200–700 µm), having sufficient intracellular space to contain an exceptionally high number of chromosomes (Angert et al. 1993). *Synechocystis* sp. PCC 6803, in contrast, is similar in size (2 µm) to *T. kodakarensis*, and one may wonder how it is able to accommodate up to 10 times more chromosomes (Atomi et al. 2004; Lea-Smith et al. 2014).

The finding that *T. kodakarensis* is polyploid is also in good agreement with results obtained in chromosome quantification studies of the genetically accessible extremophiles *D. radiodurans* and *T. thermophilus* (Cao et al. 2010; Ohtani et al. 2010). Both bacteria were reported to have similar engineering difficulties as the ones occasionally observed for *T. kodakarensis* and were subsequently found to have multiple copies (6–10 and 4–5, respectively) of their chromosome. The existence of multiple chromosome copies can readily explain the occasionally observed difficulty in obtaining homozygous recombinant cultures, as it entails that the desired strain is only obtained when the modification is incorporated in all chromosome copies.

### Physical implications of polyploidy

Based on the results obtained, several questions arise: (1) how are polyploid prokaryotes able to fit that many copies of its chromosome in a cell, and related to that, how is spontaneous aggregation due to the high DNA concentrations prevented, and (2) what possible evolutionary advantages could polyploidy have? Since the latter aspect has been thoroughly covered in recent papers (Soppa 2013; Zerulla et al. 2014; Zerulla and Soppa 2014), describing potential advantages such as conferring resistance to DNA damage or allowing heterozygosity to occur (both of which might be beneficial for survival in extreme conditions), only the former question will be briefly addressed here.

It is well known that all living things have to extensively condense their genomic DNA to fit it in the physically small space of a cell. In addition, this so-called DNA packaging also prevents spontaneous aggregation due to the high concentration imposed by the confinement (Minsky et al. 1997). The key mechanisms employed for DNA packaging are DNA supercoiling, macromolecular crowding and the association of DNA-binding proteins that fold the genome into a more condensed structure. The interactions between DNA-binding proteins and the genomic DNA can have different structural effects on DNA, such as bending, bridging or wrapping, each of which mediates DNA packaging in a different way [reviewed in e.g., (Luijsterburg et al. 2008; Sandman et al. 1998)].

DNA-binding proteins are generally small basic proteins that are abundantly present. The best examples are the eukaryotic histones that wrap genomic DNA by folding it around their surface forming so-called nucleosomes, the primary unit of DNA compaction. Archaeal homologs of histone proteins have been found in almost all Euryarchaeota (including *T. kodakarensis*), Nanoarchaeota and Thaumarchaeota, but are generally not encoded by Crenarchaeota (Čuboňová et al. 2012; Higashibata et al. 1999; Maruyama et al. 2013, 2011; Sandman and Reeve 2006; White and Bell 2002). Interestingly, a similar dichotomy between Euryarchaeota and Crenarchaeota can be observed regarding their ploidy levels: almost all tested Euryarchaeal species have been found to be polyploid, while all the Crenarchaeal species tested to date were found to be monoploid (Hildenbrand et al. 2011) (Table 1). Whether there is any correlation between these observations is currently unknown, however, it is tempting to speculate that histones play an important role in enabling polyploidy in Archaea.

While DNA packaging explains how a single genome is sufficiently condensed to fit the physically small space of a cell, it does not clarify how many chromosome copies actually fit in. To answer this question we compared the situation in *T. kodakarensis* with the situation in *E. coli*, for which it is known that on average two chromosome copies [~9.2 Mbp in total, ~0.136–0.16 µm^3^ (Odijk 1998)] occupy about a quarter of the total cell volume [total cell volume of *E.* coli: 0.58–0.69 µm^3^ (Kubitschek 1990)] (Dame 2005). Assuming that DNA packaging in *T. kodakarensis* is as efficient as in *E. coli* and that the irregular cocci of *T. kodakarensis* are perfect spheres (having a cell diameter of 1–2 µm and hence a cell volume of ~0.52–4.18 µm^3^), fitting up to ~19 copies of its chromosome (~39.7 Mbp in total, ~0.587–0.690 µm^3^), should physically, thus, be possible.

## Conclusion

We clearly showed that *T. kodakarensis* has multiple chromosome copies and that this result supports earlier reports on the presence of polyploidy in Euryarchaeota. The existence of multiple chromosome copies can also readily explain the occasionally observed difficulty in obtaining homozygous recombinant cultures, as it entails that the desired strain is only acquired when the modification is incorporated in all chromosome copies. Moreover, an apparent correlation between the presence of histones and polyploidy in Archaea is observed. The histones may assist in efficient DNA packaging which is required for fitting multiple chromosomes in a single cell.

## Electronic supplementary material

Supplementary material 1 (XLSX 191 kb)

Supplementary material 2 (DOCX 12 kb)
