# Supplementary material for: The chromosome copy number of the hyperthermophilic archaeon Thermococcus kodakarensis KOD1
Source: Extremophiles. 2015 May 8;19(4):741–50. doi: 10.1007/s00792-015-0750-5 (PMC4502288; doi:10.1007/s00792-015-0750-5)
Supplement: Supplementary file 2 — Supplementary material 2 (DOCX 12 kb) [file 792_2015_750_MOESM2_ESM.docx]

| Oligonucleotide | Sequence (5’-3’) | Application |
| --- | --- | --- |
| BG5059 | AAGCTACCTCGACCAGTATC | Synthesis of real-time PCR standard; 1 kb fragment internal to TK1765 |
| BG5060 | GTCCCAGTAGTCCATAACTC | Synthesis of real-time PCR standard; 1 kb fragment internal to TK1765 |
| BG5329 | ACAAGGACTACCTCCTAACG | Detection of genome copies in *T. kodakarensis*, primer set 1 (300 bp fragment) |
| BG5330 | CAAAGCTCCTGCTGTAGAAG | Detection of genome copies in *T. kodakarensis*, primer set 1 (300 bp fragment) |
| BG5331 | ACTACGCCTTCCTCGACCTC | Detection of genome copies in *T. kodakarensis*, primer set 2 (293 bp fragment) |
| BG5332 | GTAGTTGCCCGCCATACCTC | Detection of genome copies in *T. kodakarensis*, primer set 2 (293 bp fragment) |
| BG5333 | AGAGTCCCGACGACGGTAAG | Detection of genome copies in *T. kodakarensis*, primer set 3 (306 bp fragment) |
| BG5334 | CGGTGTAGTTGACGCAGAAG | Detection of genome copies in *T. kodakarensis*, primer set 3 (306 bp fragment) |
